# 1,25D3 prevents CD8^+^Tc2 skewing and asthma development through VDR binding changes to the *Cyp11a1* promoter

**DOI:** 10.1038/ncomms10213

**Published:** 2016-01-11

**Authors:** Michaela Schedel, Yi Jia, Sven Michel, Katsuyuki Takeda, Joanne Domenico, Anthony Joetham, Fangkun Ning, Matthew Strand, Junyan Han, Meiqin Wang, Joseph J. Lucas, Christian Vogelberg, Michael Kabesch, Brian P. O’Connor, Erwin W. Gelfand

**Affiliations:** 1 Division of Cell Biology, Department of Pediatrics, National Jewish Health, 1400 Jackson Street, Denver, Colorado 80206, USA; 2 University Children’s Hospital Regensburg (KUNO), Department of Pediatric Pneumology and Allergy, Steinmetzstrasse 1-3, 93049 Regensburg, Germany; 3 Department of Pediatric Pneumology, Allergy and Neonatology, Hannover Medical School, Carl-Neuberg-Strasse, 30625 Hannover, Germany; 4 Division of Biostatistics and Bioinformatics, National Jewish Health, 1400 Jackson Street, Denver, Colorado 80206, USA; 5 Department of Pediatrics, University Children’s Hospital, Technical University, Fetscherstraße 74, 01307 Dresden, Germany; 6 Center for Genes, Environment and Health, National Jewish Health, 1400 Jackson Street, Denver, Colorado 80206, USA; 7 Department of Immunology and Microbiology, University of Colorado AMC, 13001 E 17th Place, Aurora, Colorado 80045, USA

## Abstract

Effector CD8^+^ T cells convert from IFN-γ^+^ (Tc1) to IL-13^+^ (Tc2) cells in the presence of IL-4. Underlying regulatory mechanisms are not fully defined. Here, we show that addition of 1,25D3, the active form of vitamin D3, during CD8^+^ T-cell differentiation prevents IL-4-induced conversion to IL-13-producers. Transfer of 1,25D3-treated CD8^+^ T cells into sensitized and challenged CD8^+^-deficient recipients fails to restore development of lung allergic responses. 1,25D3 alters vitamin D receptor (VDR) recruitment to the *Cyp11a1* promoter *in vitro* and *in vivo* in the presence of IL-4. As a result, protein levels and enzymatic activity of CYP11A1, a steroidogenic enzyme regulating CD8^+^ T-cell conversion, are decreased. An epistatic effect between *CYP11A1* and *VDR* polymorphisms may contribute to the predisposition to childhood asthma. These data identify a role for 1,25D3 in the molecular programming of CD8^+^ T-cell conversion to an IL-13-secreting phenotype through regulation of steroidogenesis, potentially governing asthma susceptibility.

## Abstract (web summary)

Type 2 CD8^+^ T cells (Tc2) play a role in the development of experimental asthma. Here the authors show that 1,25D3, the active form of vitamin D3, can prevent conversion of CD8^+^T cells to a Tc2 phenotype, reducing asthma susceptibility.

## Graphical abstract

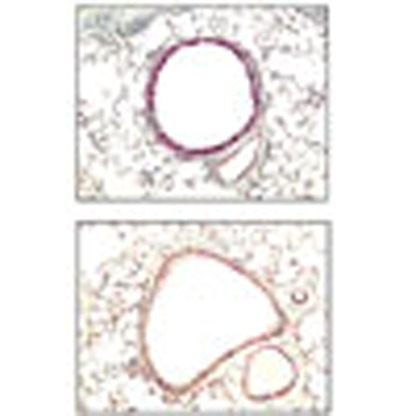

For a large percentage of asthmatics, inhaled corticosteroids are the most effective first-line treatment to control airway inflammation and symptoms in persistent asthma, but an estimated 40% of asthmatics who fail to respond to corticosteroid show no improvement in airway function^1^. Hence, steroid-refractory asthma remains a clinical challenge. We and others have demonstrated an important role for type 2 (Tc2) CD8^+^ T cells in the development of experimental asthma^2^,^3^,^4^,^5^,^6^,^7^,^8^,^9^ as a result of their activation by IL-4-producing CD4^+^ T cells^10^. In humans, increased numbers of CD8^+^ T cells, which are more resistant than CD4^+^ T cells to corticosteroids^11^, have been detected in steroid-refractory asthmatics^12^ and correlated with lower lung function and reticular basement membrane thickening^13^.

Over the last decade, deficiency in vitamin D, a member of the steroid family, has been associated with various inflammatory diseases^14^,^15^,^16^,^17^ including steroid-refractory asthma^18^,^19^. An association between lower levels of vitamin D and increased asthma severity, reduced lung function and poor asthma control has been suggested^19^,^20^,^21^,^22^,^23^,^24^,^25^. However, it is unclear if vitamin D supplementation impacts the disease as seen in a recent trial in asthmatics^26^ but a potential mechanism of action remains unknown.

Previously, we identified CYP11A1 as an essential component of a novel, pro-allergic mechanistic axis in the development of experimental asthma (CD8^+^ T cells)^4^,^27^ and peanut-induced allergy (CD4^+^ T cells)^28^. CYP11A1, a mitochondrial P450 cytochrome, is the first and rate-limiting enzyme in steroidogenesis converting cholesterol to pregnenolone^29^. In the presence of IL-4, CYP11A1 was identified as a critical regulator of CD8^+^ T-cell conversion. Together with antigen receptor signalling of differentiated CD8^+^ T cells, CYP11A1 activation was essential for increased IL-13 and decreased IFN-γ production^4^,^27^. These data linked for the first time steroidogenesis in CD8^+^ T cells, a non-classical steroidogenic tissue, to a pro-allergic differentiation pathway.

In this study, we demonstrate the role of 1,25D3 as a key modulator of the functional conversion of CD8^+^ T cells from IFN-γ- to IL-13-producing cells via a mechanistic link to CYP11A1 activity. This effect appears driven by 1,25D3-mediated changes in the recruitment of the VDR transcription factor to the promoter region of *Cyp11a1* paralleled by changes in the enzymatic activation of CYP11A1 and the prevention of lung allergic responses. An epistasic effect between genetic variants in *CYP11A1* and *VDR* is implicated in humans due to protective effects on the development of asthma.

## Results

### 1,25D3 prevents conversion to IL-13-producing CD8^+^ T cells

**Figure 1:**
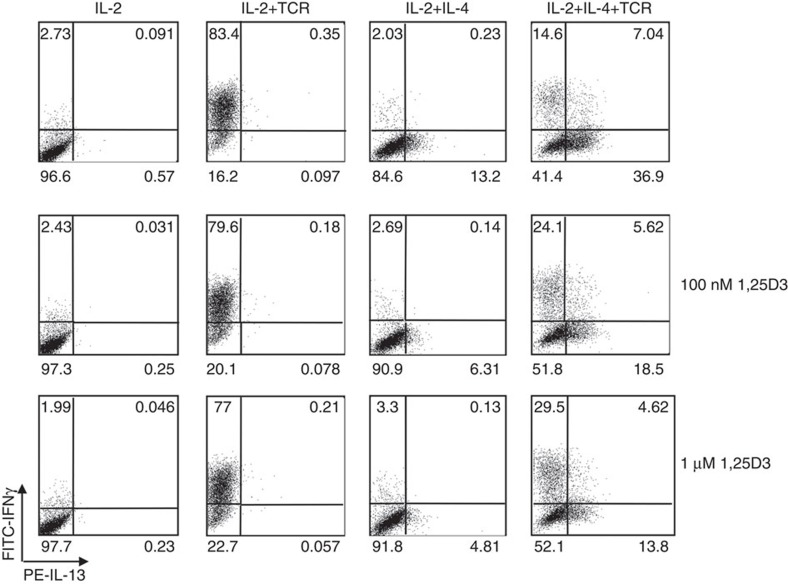
IFN-γ and IL-13 expression in CD8^+^ T cells differentiated in IL-2 or IL-2+IL-4 in the presence or absence of 1,25D3 at 100 nM or 1 μM. Representative results of intracellular staining of IFN-γ and IL-13 expression in CD8^+^ T cells with or without SIINFEKL (T-cell receptor, TCR) restimulation.

We previously demonstrated that in the presence of IL-4, CD8^+^ T cells convert from IFN-γ CD8^+^ effector T cells to pathogenic IL-13 producers, triggering the full spectrum of lung allergic responses^4^,^27^. To investigate the effects of vitamin D on this functional conversion of CD8^+^ T cells, the active form of vitamin D, 1,25(OH)_2_D_3_ (further referred to as 1,25D3, 100 nM, 1 μM), is added during cell differentiation. 1,25D3 has no significant effect on cell viability (Supplementary Fig. 1). When CD8^+^ T cells are cultured with SIINFEKL and IL-2+IL-4 in the presence of 1,25D3, a dose-dependent decrease in the percentage of IL-13^+^ cells and an increase in IFN-γ^+^ cells is observed (Fig. 1). After adding 100 nM 1,25D3, IL-13-single-positive cells decrease from 23.8±9.3 (mean±s.e.m.) to 11.3±4.8%, whereas IFN-γ-single-positive cells increase from 16.8±5.6 to 24.5±4.8% (Fig. 1, Supplementary Table 1). This effect is even more pronounced after culture with 1 μM 1,25D3 (Fig. 1, Supplementary Table 1).

When 1,25D3 is added during the antigen (SIINFEKL) re-stimulation phase in the last 4 h of culture, the cytokine profiles of differentiated CD8^+^ T cells generated in the presence of IL-2+IL-4 and 100 nM or 1 μM of the drug are unaffected (Supplementary Fig. 2a,b). These results suggest a significant role for 1,25D3 only during the conversion of CD8^+^ T cells in an IL-4-rich environment but not on differentiated cells.

### 1,25D3 alters functional activity of CYP11A1 in CD8^+^ T cells

**Figure 2:**
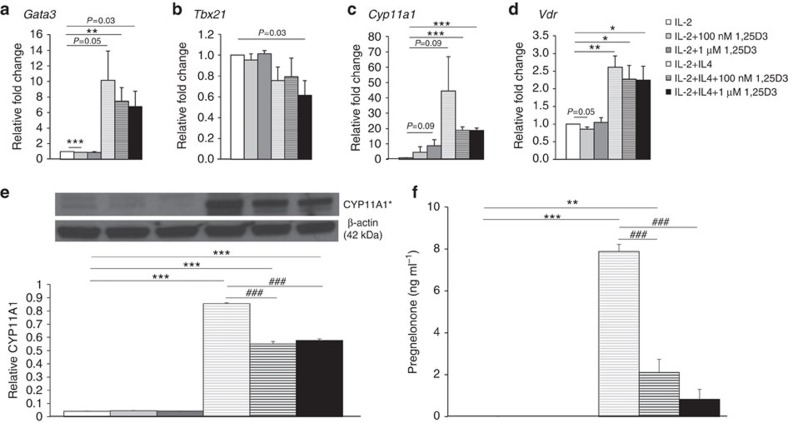
1,25D3 treatment of CD8^+^ T cells alters gene expression of transcription factors and the functional activity of CYP11A1. Gene expression of (**a**) *Gata3*, (**b**) *Tbx21*, (**c**) *Cyp11a1* and (**d**) *Vdr* was measured by quantitative PCR (qPCR) in CD8^+^ T cells in IL-2 or IL-2+IL-4 in the presence or absence of 100 nM or 1 μM 1,25D3. Results (relative fold change+s.e.m.) are from three independent experiments. (**e**) CYP11A1 protein levels (mean+s.e.m.) detected by immunoblot analyses and densitometry of autoradiographs in CD8^+^ T cells differentiated in IL-2 or IL-2+IL-4 in the presence or absence of 100 nM or 1 μM 1,25D3. Results are from three independent experiments, * Denotes calculated molecular weight (MW) for CYP11A1 (13363-1-AP, Proteintech, Chicago, IL): 60 kDa, observed MW: 49 kDa, (**f**) Pregnenolone levels (mean+s.e.m.) determined by ELISA in supernatants from CD8^+^ T cells differentiated in IL-2 or IL-2+IL-4 in the presence or absence of 100 nM or 1 μM 1,25D3. Results are from six independent experiments. Linear mixed models were employed; pairwise comparisons were performed using *t-tests* derived from these models. *P<0.05, **P<0.01, ***P<0.001 compared with the IL-2 group, ###*P*<0.001 compared with the IL-2+IL-4 group, these *P* values remained significant after correction for multiple comparisons (Benjamini–Hochberg^58^ correction); *P* values that did not reach the threshold *P* value after adjustment for multiple comparisons are shown numerically.

The major transcription factors, *Tbx21* and *Gata3*, regulate expression of IFN-γ and IL-13 in T cells^30^. During the differentiation of CD8^+^ T cells with IL-2+IL-4, we observe decreased *Tbx21* mRNA levels while *Gata3* gene expression is increased compared with cells differentiated in IL-2 alone (Fig. 2a,b).

We previously demonstrated that the enzymatic activation of CYP11A1 plays a key role in the phenotypic conversion of CD8^+^ T cells from IFN-γ- to IL-13-producing cells^4^,^27^. A similar mechanism during the stages of CD8^+^ T-cell differentiation in the presence of 1,25D3 is postulated. CYP11A1 transcript (Fig. 2c) and protein (Fig. 2e, Supplementary Fig. 3) levels are decreased in the presence of 1,25D3 (100 nM or 1 μM) during the differentiation of CD8^+^ T cells in IL-2+IL-4 to a greater extent than the lineage-specific transcription factors *Gata3* and *Tbx21*. In parallel, decreased levels of pregnenolone are detected in supernatants from cells cultured in the presence of 1,25D3 (Fig. 2f). These data strengthen the notion that in the presence of 1,25D3, CYP11A1 may be a functional regulator of the conversion of CD8^+^ T cells from IFN-γ- to IL-13-producing cells. Of note, in CD8^+^ T cells differentiated in IL-2+IL-4, *Vdr* gene expression is significantly enhanced while additionally adding 1,25D3 during the differentiation process reveals a trend towards lower *Vdr* levels (Fig. 2d).

### 1,25D3 alters VDR binding to the *Cyp11a1* promoter

**Figure 3:**
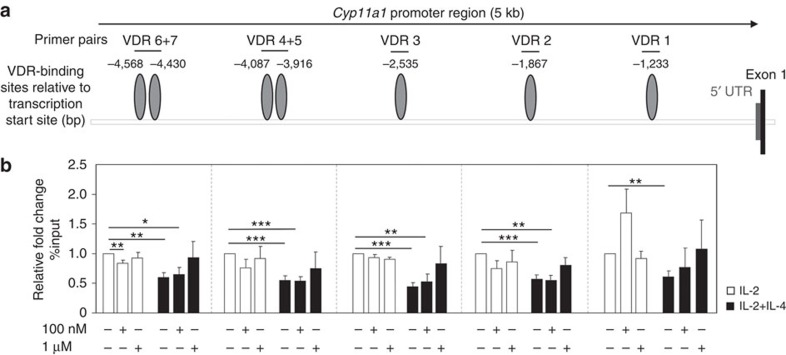
1,25D3-mediated VDR recruitment to the *Cyp11a1* promoter region is altered in CD8^+^ T cells differentiated in IL-2 or IL-2+IL-4 in the presence or absence of 100 nM or 1 μM 1,25D3. (**a**) Localization of VDR-binding sites and qPCR primers in the *Cyp11a1* promoter region. (**b**) qPCR was performed using five *Cyp11a1* promoter-specific primers covering seven VDR-binding sites. Data were analysed via the percent input methodology: (2^CT of total input−CT of specific IP^) × 100 and relative percent input ratios using CD8^+^ T cells stimulated with IL-2 as baseline. Data (relative fold change+s.e.m.) are from three independent experiments. Linear mixed models were employed; pairwise comparisons were performed using *t*-tests derived from these models. *P<0.05, **P<0.01, ***P<0.001 compared with the IL-2 group, these *P* values remained significant after correction for multiple comparisons; (Benjamini–Hochberg^58^ correction). No statistical significance was detected compared with the IL-2+IL-4 group.

1,25D3 primarily mediates signals in the cell through the transcription factor vitamin D receptor (VDR), which regulates the transcriptional activity of many target genes^31^,^32^. *In silico* transcription factor-binding analyses of the *Cyp11a1* promoter region (5 kb) predict seven potential VDR-binding sites (Supplementary Fig. 4). To define the molecular mechanism underlying the 1,25D3-mediated prevention of the conversion of CD8^+^ T cells driven by CYP11A1, we evaluate recruitment of VDR to the *Cyp11a1* promoter region via chromatin immunoprecipitation (ChIP). In CD8^+^ T cells cultured in IL-2 or IL-2+IL-4 in the absence or presence of 1,25D3 (100 nM or 1 μM), the relative abundance of *Cyp11a1* promoter DNA immunoprecipitated by a VDR-specific antibody is determined by quantitative PCR using five *Cyp11a1*-specific primer pairs (Supplementary Fig. 4, Supplementary Table 5). Due to the close vicinity of some of the predicted VDR-binding sites, primers cover multiple potential VDR-binding sites (for example, primer pairs 4 and 5, Supplementary Fig. 4). A representative experiment of the percent input immunoprecipitated by the VDR and the negative control IgG antibody is presented in the Supplementary Fig. 5a–e. Considering the complexity of the nuclear environment in primary CD8^+^ T cells, normalization between three independent experiments is performed via the percent input methodology and the relative percent input ratios are displayed using CD8^+^ T cells stimulated with IL-2 as baseline (see more details in Methods). The data show that in the presence of IL-4 (black bars, Fig. 3b), the recruitment of VDR to the *Cyp11a1* promoter region is significantly reduced for all VDR-binding sites in comparison to CD8^+^ T cells stimulated with IL-2 alone (white bars, Fig. 3b). Intriguingly, CD8^+^ T cells cultured in IL-2+IL-4 together with 1,25D3 (1 μM) increase the association of VDR to *Cyp11a1*, almost to levels seen in IL-2-differentiated CD8^+^ T cells at baseline. In contrast, 1,25D3 has almost no effect on the VDR recruitment in CD8^+^ T cells after IL-2 stimulation alone. These results suggest that VDR binding to the *Cyp11a1* promoter acts as a transcriptional repressor since the reduction in VDR recruitment to its promoter leads to increased CYP11A1 gene and protein expression (Fig. 2c,e) and elevated pregnenolone levels (Fig. 2f).

### 1,25D3-treated CD8^+^ T cells prevent lung allergic responses

**Figure 4:**
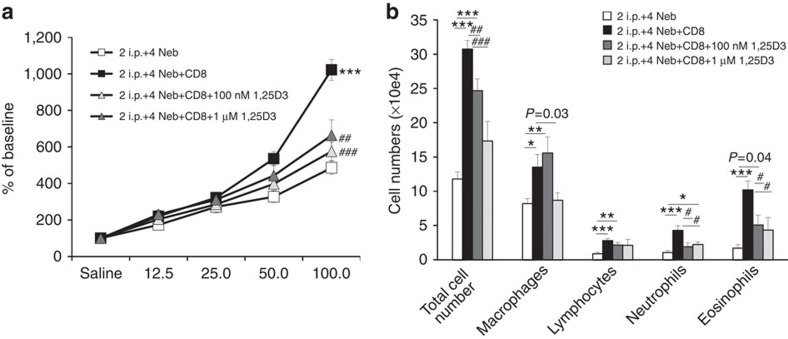
Adoptive transfer of 1,25D3-treated CD8^+^ T cells into CD8-deficient recipients fails to induce AHR. Recipient mice were sensitized (two intraperitoneal, i.p.) and challenged (four nebulizations, Neb) using secondary allergen challenge model and received no cells, CD8^+^ T cells differentiated in IL-2 alone (CD8) or in the presence of 100 nM (CD8+100 nM 1,25D3) or 1 μM 1,25D3 (CD8+1 μM 1,25D3). (**a**) Changes in airway resistance (RL) were measured in response to increasing concentrations of methacholine. (**b**) Cell composition in BAL fluid. Data (mean+s.e.m.) are from two to three experiments with three to four mice per experiment. *P<0.05, **P<0.01, ***P<0.001; compared with sensitized and challenged CD8-deficient recipients that received no cells. General linear models were employed; pairwise comparisons were performed using *t*-tests derived from these models. #*P*<0.05, ##*P*<0.01, ###*P*<0.001 compared with sensitized and challenged CD8-deficient recipients that received CD8^+^ T cells differentiated in IL-2 alone, these *P* values remained significant after correction for multiple comparisons (Benjamini–Hochberg^58^ correction); *P* values that did not reach the threshold *P* value after adjustment for multiple comparisons are shown numerically.

**Figure 5:**
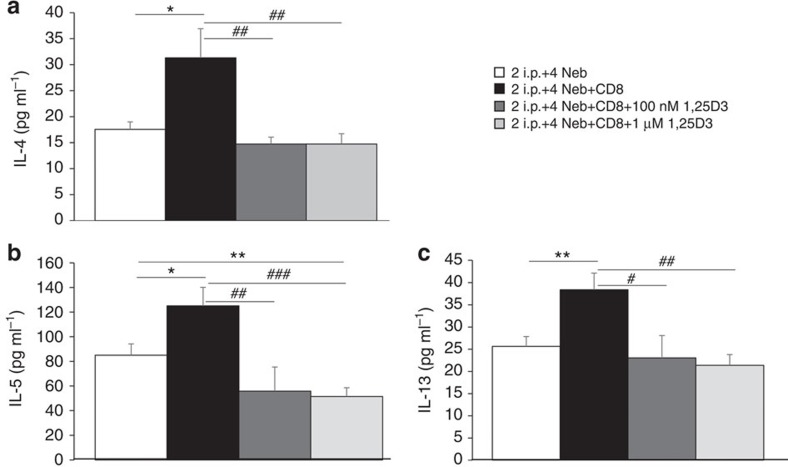
Adoptive transfer of 1,25D3-treated CD8^+^ T cells into CD8-deficient recipients decreased cytokine levels in the BAL. Cytokine levels in BAL fluid (mean+s.e.m.): (**a**) IL-4, (**b**) IL-5 and (**c**) IL-13. *P<0.05, **P<0.01; compared with sensitized and challenged CD8-deficient recipients that received no cells. Data (mean+s.e.m.) are from two to three experiments with three to four mice per experiment. General linear models were employed; pairwise comparisons were performed using *t*-tests derived from these models. #*P*<0.05, ##*P*<0.01, ###*P*<0.001 compared with sensitized and challenged CD8-deficient recipients that received CD8^+^ T cells differentiated in IL-2 alone, *P* values remained significant after correction for multiple comparisons (Benjamini–Hochberg^58^ correction).

**Figure 6:**
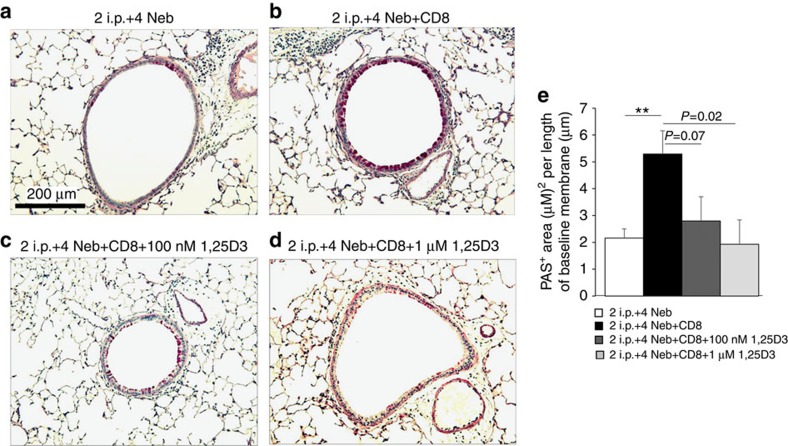
Adoptive transfer of 1,25D3-treated CD8^+^ T cells into CD8-deficient recipients prevents goblet cell metaplasia. (**a**–**d**) Representative photomicrographs of lung histology (original magnification × 3,200, scale bar, 200 μm). (**e**) Quantitative analysis of PAS-positive goblet cells was determined in cross-sectional areas of the airway wall. Data (mean+s.e.m.) are from two experiments with three mice per experiment. Linear mixed models were employed; pairwise comparisons were performed using *t*-tests derived from these models. **P<0.01 these *P* values remained significant after correction for multiple comparisons; *P* values that did not reach the threshold *P* value after adjustment for multiple comparisons (Benjamini–Hochberg^58^ correction) are shown numerically.

Since 1,25D3 prevents the IL-4-induced conversion of CD8^+^ T cells from IFN-γ to IL-13 production *in vitro*, we examine if 1,25D3 treatment of CD8^+^ T cells attenuates restoration of lung allergic responses following adoptive transfer *in vivo* into CD8-deficient mice. Adoptive transfer of IL-2-differentiated CD8^+^ T cells into sensitized and challenged CD8-deficient recipients followed by secondary allergen challenge fully restores lung allergic responses (Fig. 4a,b). In contrast, transfer of IL-2-differentiated CD8^+^ T cells cultured in the presence of 1,25D3 (100 nM or 1 μM) fails to restore airway hyperresponsiveness (AHR; Fig. 4a) or airway inflammation (Fig. 4b). Levels of IL-4, IL-5 and IL-13 are significantly lower in the bronchoalveolar lavage (BAL) fluid of these recipient mice compared with recipients of untreated cells (Fig. 5a–c). Lung sections confirm that these recipients show less inflammation and significantly decreased numbers of PAS^+^ mucus-containing goblet cells (Fig. 6a–e).

**Figure 7:**
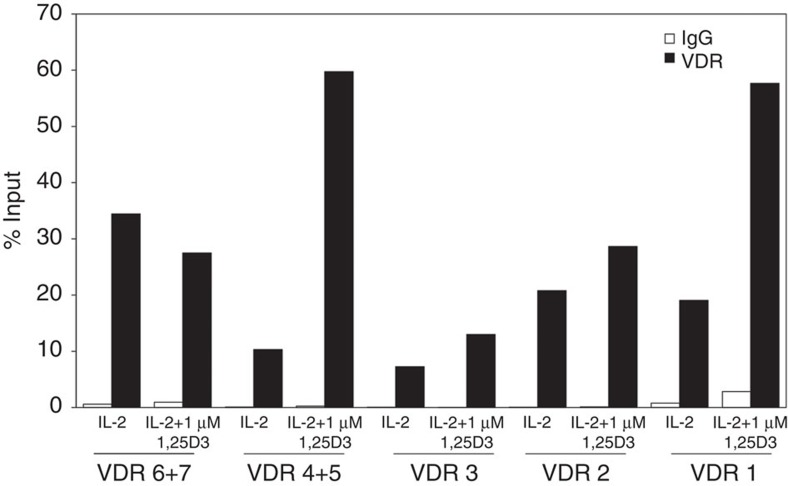
1,25D3-mediated VDR recruitment to the *Cyp11a1* promoter region is altered in CD8^+^ T cells after adoptive transfer into sensitized and challenged CD8-deficient mice. CD8^+^ T cells were differentiated *in vitro* in IL-2 in the presence or absence of 1 μM 1,25D3. CD8^+^ T cells from the lungs were recovered after adoptive transfer into sensitized and challenged CD8-deficient mice (*n*=3 mice per group). qPCR was performed using five *Cyp11a1* promoter-specific primers covering seven VDR-binding sites. Data were analysed via the percent input methodology: (2^CT of total input−CT of specific IP^) × 100.

To confirm that the observed effects are dependent on the binding of VDR to the *Cyp11a1* promoter region, adoptively transferred CD8^+^ T cells are isolated from the lungs of ovalbumin (OVA)-sensitized mice after allergen challenge followed by ChIP experiments. As seen above in the *in vitro* experiments, isolated 1,25D3-treated CD8^+^ T cells from the lungs of sensitized and challenged recipients show increased recruitment of VDR to the *Cyp11a1* promoter region in comparison to recipients of untreated CD8^+^ T cells differentiated in IL-2 alone for four out of the five VDR-binding sites under study (Fig. 7). These *in vivo* results together with the *in vitro* findings support the hypothesis that CYP11A1 plays a key role in both preventing the conversion of CD8^+^ T cells from IFN-γ- to IL-13-producers in the presence of 1,25D3 and the failure to induce AHR is the result of 1,25D3-triggered VDR binding to the *Cyp11a1* promoter region.

### Genetic asthma predisposition of *CYP11A1* in children

Given the importance of CYP11A1 in the regulation of experimental asthma in mice, we determine if a predisposition to asthma in humans is influenced by *CYP11A1* single-nucleotide polymorphisms (SNPs). The 25 polymorphisms in *CYP11A1* cluster in five tagging bins and three single SNPs are identified (Supplementary Fig. 6 and Supplementary Table 2). A significant protective effect in asthma is observed for three (rs4886595, rs4432229 and rs11632698) out of eight tested SNPs in the case–control population with an odds ratio (OR) ranging from 0.77 to 0.85 (Table 1). Association results for SNP rs4886595 remains significant after correction for multiple testing (Table 1). Seven out of eight polymorphisms covered by the asthma-associated tagging SNPs are located in potential regulatory regions of *CYP11A1* (promoter: *N*=6, introns: *N*=1). Allele-specific *in silico* analyses predict changes in transcription factor binding for all of these SNPs (Supplementary Table 2). Computed score-based analyses of multiple high-throughput datasets (RegulomeDB Database, Supplementary Table 3) suggest a putative regulatory role of some of the asthma-associated *CYP11A1* SNPs. Interestingly, for polymorphism rs8039957 (tagging bin 1, covered by rs4432229) located in the *CYP11A1* promoter region, a novel VDR-binding site is predicted in the presence of the polymorphic allele. Three *CYP11A1* SNPs within this block and rs4886595 reveal allele-specific alterations on *CYP11A1* gene expression (Supplementary Table 3).

Because of these findings and the observed transcriptional regulation of *Cyp11a1* by VDR in mice, a combined analysis of SNPs in *CYP11A1* and *VDR* is performed to further determine if an epistatic phenomenon, here defined as the effect of one locus being dependent on the genotype of a second locus, may be involved in the development of childhood asthma. The most significant asthma-associated SNPs in our population for *VDR* rs2107301 (ref. 33) and *CYP11A1* rs4886595 are selected for the analyses. In addition, *CYP11A1* SNP rs4432229 is included, since rs8039957 which is within the same tagging bin, is predicted to affect allele-specific binding of VDR to the *CYP11A1* promoter region. Epistasis is observed when the effects of the respective protective alleles in *CYP11A1* or *VDR* are studied after stratification for individuals carrying the corresponding SNP located in the other gene (Table 2). Both *CYP11A1* SNPs, rs4886595 and rs4432229, are significantly associated with asthma only in individuals carrying homozygous asthma-risk alleles of rs2107301 (*VDR*). Thus, *CYP11A1* polymorphisms may alter the pro-allergic function of CYP11A1 specifically in combination with modifier SNPs in *VDR*.

## Discussion

Although cluster designations have identified some of the clinical features in asthmatics, actual definition of underlying pathobiology has lagged behind, thus limiting targeted therapy. To date, only one endotype has been well-characterized, a T_H_2-high-signature associated with CD4^+^ T cells, IL-13, and steroid responsiveness. There has been accumulating evidence for the role of CD8^+^ T cells in asthma, especially in steroid-refractory asthma^2^,^3^,^4^,^5^,^6^,^7^,^8^,^9^,^11^,^12^,^13^,^34^. We have shown *in vitro* and *in vivo* that in the presence of IL-4, mouse CD8^+^ T cells can convert from IFN-γ producers to a significant source of IL-13 (refs 4, 27). This conversion was associated with changes in lineage-specific transcription factor signatures and histone modifications at key loci. In the terminal stage of differentiation to IL-13 production, the enzymatic activation of CYP11A1 was shown to play an essential role^4^,^27^. Since vitamin D deficiency has been implicated in refractory asthma, although without a defined molecular mechanism, we examined if 1,25D3 modulated the conversion of IL-13^+^CD8^+^ T cells, a pathway shown to play a role in asthma^4^,^27^. We now demonstrate that differentiation of CD8^+^ T cells in the presence of 1,25D3 prevented the IL-4-mediated skewing of CD8^+^ T cells to IL-13-producing Tc2 cells. As a consequence, adoptively transferred CD8^+^ T cells, differentiated in the presence of 1,25D3, failed to induce lung allergic responses in sensitized and challenged CD8-deficient recipients. This failure appeared linked to 1,25D3 induced changes in the binding of the VDR transcription factor to the *Cyp11a1* promoter region. In humans, the data supported an epistatic effect between *CYP11A1* and *VDR* polymorphisms, diminishing the risk to develop childhood asthma.

Vitamin D has previously been shown to play a critical role in modulating effects of CD4^+^ T-cell subsets^35^,^36^,^37^ and even enhanced the therapeutic response(s) to corticosteroids^38^. However, little mechanistic data are available on the impact of 1,25D3 on CD8^+^ T cells. When 1,25D3 was added during CD8^+^ T-cell differentiation, the IFN-γ-producing capacity of Tc1 cells differentiated in IL-2 alone was not affected. However, the cytokine profile in CD8^+^ T cells differentiated in IL-2+IL-4 was significantly modified, characterized by increased IFN-γ and decreased IL-13 production. Our findings suggested that the activity of 1,25D3 was dependent on the exposure to IL-4 during CD8^+^ T-cell skewing, which can mediate the epigenetic poising of CD8^+^ T cells at loci sensitive to VDR activity. Thus, it is possible that fully differentiated CD8^+^ Tc2 cells are insensitive to 1,25D3 due to previous changes in chromatin structure at critical loci, now insensitive to VDR.

In light of the immunomodulatory activities associated with vitamin D^14^,^39^,^40^, there has been increased interest in determining its role in asthma. However, results of clinical trials using vitamin D as a therapeutic supplement^19^,^20^,^21^,^22^,^24^,^26^,^41^,^42^,^43^ or during pregnancy to attenuate development of atopic disease in progeny^44^,^45^,^46^,^47^,^48^,^49^ have been conflicting. Our findings suggest that vitamin D supplementation may exhibit preventive activities rather than increase the therapeutic potential during an asthma exacerbation as the molecular mechanisms implicated in responsive cells at that stage may have already been epigenetically poised; however, by modifying transcription of key pro-allergic transcription factor expression an early stage may attenuate disease progression. Further, vitamin D as an interventional strategy may be restricted to a well-defined subpopulation of steroid-refractory asthmatics with increased numbers of CD8^+^IL-13^+^ T cells (Tc2 cells).

During differentiation of CD8^+^ T cells in the presence of IL-4 and 1,25D3, lineage-specific markers (for example, *Gata3* and *Tbx21*) were only marginally altered, whereas CYP11A1 gene and protein expression were significantly affected in a dose-dependent manner. Changes at a transcriptomic level appeared weaker, likely the result of analysis on day 4 of the CD8^+^ T-cell differentiation protocol. Importantly, the enzymatic activity of CYP11A1, as measured by generation of pregnenolone, was markedly reduced. Together with T-cell receptor engagement, the relevance of this essential factor in the conversion of CD8^+^ T cells to IL-13 producers was augmented. 1,25D3 was only effective when included during CD8^+^ differentiation and was without effect when added during the terminal differentiation stage.

Adoptive transfer of 1,25D3-treated CD8^+^ T cells into sensitized and challenged CD8-deficient mice failed to induce lung allergic responses. Similar inhibitor*y* effects on conversion were observed following inhibition of the enzymatic activity of CYP11A1 (with aminoglutethimide) or silencing of the gene^4^ as both were associated with the failure to restore lung allergic responses in sensitized and challenged CD8-deficient mice. These results indicated that 1,25D3, similar to aminoglutethimide, acts upstream of CYP11A1. The effects on mouse CD8^+^ T cells required 100 nM to 1 μM concentrations of 1,25D3, concentration (500 nM calcitriol) previously demonstrated *in vitro* to be effective in CD4^+^ T cells from steroid-resistant asthmatics^50^. These results suggest that *in vitro*, higher concentrations of 1,25D3 are needed to mimic potential *in vivo* effects.

Little is known about molecular mechanisms mediated by 1,25D3 in CD8^+^ T cells, specifically in the conversion to IL-13 production and contributions to asthma pathogenesis. The active form of vitamin D primarily transmits a signal through the transcription factor VDR, a member of the nuclear hormone receptor family^51^. By performing ChIP experiments with a VDR-specific antibody, we showed that VDR potentially acts as a transcriptional repressor of CYP11A1; in IL-4-activated CD8^+^ T cells, the recruitment of VDR to *Cyp11a1* was decreased leading to higher CYP11A1 expression, whereas in CD8^+^ cells cultured in IL-2 alone, more VDR binding paralleled by lower CYP11A1 expression was detected. A similar effect on IL-12B expression has previously been reported in lipopolysaccharide-treated human monocytes (THP-1)^52^. Vitamin D induced alterations in the recruitment of VDR/RXR, the co-repressor NCOR2/SMRT, and histone deacetylase 3 paralleled by decreased histone 4 acetylation (H4ac), and increased histone 3 trimethylation (H3K27me3), potentially causing the downregulation of IL-12B. In the CD8^+^ T-cell differentiation model investigated here, we propose that 1,25D3-induced recruitment of VDR to the *Cyp11a1* promoter region results in lower CYP11A1 enzymatic activity, which in turn, is associated with attenuation of the IL-4-driven conversion of CD8^+^ T cells to IL-13 production. Examination of adoptively transferred CD8^+^ T cells recovered from the lungs of sensitized and challenged recipient mice recapitulated our *in vitro* findings, as shown by the increased recruitment of VDR to the *Cyp11a1* promoter region in recovered cells from recipients of 1,25D3-treated cells. These results support the hypothesis that CYP11A1 is an important regulator of the conversion of CD8^+^ T cells and accounts for the failure of transferred 1,25D3-treated cells to restore lung allergic responses.

To determine if these findings in mice are relevant to asthma, we investigated if *CYP11A1* is involved in the susceptibility of asthma in childhood. We observed three *CYP11A1* tagging SNPs that decreased the risk for asthma in a case–control population. Even though, the effect did not reach genome-wide statistical significance, our previous findings in experimental asthma supported the relevance of CYP11A1. Most of the *CYP11A1* polymorphisms covered by the asthma-associated tagging SNPs were located in regulatory regions of *CYP11A1*, indicating a potential allele-specific function. Depending on the genotype of rs8039957, located in the *CYP11A1* promoter region, a change in VDR binding was predicted. Hence, a similar regulatory mechanism of *CYP11A1* through VDR as observed in mice may be relevant to human disease. An interaction between asthma-associated SNPs in *CYP11A1* and *VDR* supported the relevance of *CYP11A1* and *VDR* predisposing to childhood asthma. However, genetic variants in VDR have been studied extensively in the context of asthma with conflicting results^31^,^33^,^53^,^54^,^55^. To validate our findings linking asthma susceptibility to *CYP11A1* and *VDR* SNPs, the association needs to be confirmed in an independent cohort and functional studies are needed to delineate the transcriptional regulation of *CYP11A1* through VDR in humans.

In summary, the data established for the first time a mechanistic role for vitamin D in regulating the IL-4-mediated conversion of CD8^+^ T cells to IL-13-producing pathogenic effector cells. This positions a novel role for 1,25D3 as a critical regulator of the development of lung allergic responses through a unique VDR-CYP11A1-IL-13 pathway in steroid-insensitive CD8^+^ T cells. As a result, vitamin D may be beneficial in asthmatics, but restricted to a subpopulation of asthmatics characterized by the presence of these CD8^+^ T cells. Translating findings from the mouse model of experimental asthma, we identified similar mechanistic links that contribute to the susceptibility to childhood asthma. Further understanding of the molecular mechanisms by which 1,25D3, CYP11A1 and VDR interact to regulate the plasticity and/or stable conversion of CD8^+^ T cells may provide new targets and novel therapeutic strategies in asthma.

## Methods

### Animals

Pathogen free, 8–10-week-old female OT-1 mice expressing a transgenic T-cell receptor specific for SIINFEKL peptide (OVA_257–264_)^4^,^27^ and homozygous CD8-deficient mice^6^,^56^ were bred in the animal facility at National Jewish Health (Denver, CO). Studies were conducted under a protocol approved by the Institutional Animal Care and Use Committee of National Jewish Health.

### CD8^+^ T-cell culture

CD8^+^ effector memory T cells were generated *in vitro* as previously described^4^,^6^,^27^,^56^. In brief, mononuclear cells were isolated from the spleens of OT-1 mice followed by stimulation with 1 μg ml^−1^ SIINFEKL peptide for 1.5 h. Two days later, living cells were isolated using histopaque (Sigma-Aldrich, St Louis, MO) and cultured in complete RPMI 1640 medium that contained recombinant mouse IL-2 (20 ng ml^−1^, R&D, Minneapolis, MN) or IL-2+IL-4 (20 ng ml^−1^, Peprotech, Rocky Hill, NJ). For some experiments, the active form of vitamin D3, 1α,25(OH)_2_D_3_ (referred to as 1,25D3, Sigma-Aldrich) at a concentration of 100 nM or 1 μM was additionally added. Medium with cytokines was changed every day for 4 consecutive days. Cells were then re-stimulated with 1 μg ml^−1^ SIINFEKL in medium containing 2 μM monensin (Calbiochem, La Jolla, CA) for 4 h. In some experiments, 100 nM or 1 μM 1,25D3 was additionally added to the medium during the antigen re-stimulation stage.

### RNA preparation and qPCR

Total RNA was extracted from 5 × 10^6^ differentiated CD8^+^ T cells using the NucleoSpin RNA II (Macherey-Nagel, Düren, Germany) isolation kit following the manufacturer’s protocol. Total RNA (1 μg) was converted into complementary DNA using QuantiTect reverse transcription kit (Qiagen, Valencia, CA). Specific primers and probes for qPCR of *Gata3*, *Tbx21*, *Cyp11a1*, *Vdr* and the housekeeping gene *18SrRNA* were designed with Vector NTI advance10 (Life Technologies, Carlsbad, CA) and for *Tbx21* a pre-designed assay (Mm00450960_m1, LifeTechnologies) was used (Supplementary Table 4). The determined cycle threshold (CT) reflects the number of PCR cycles required for the fluorescence signal to exceed the detection threshold, which was set to the log-linear range of the amplification curve. The differences in CT values of the gene of interest and the house keeping gene *18SrRNA* were used to calculate delta CT (ΔCT). Relative fold changes were then calculated using the 2^−ΔΔCT^ algorithm.

### ELISA for pregnenolone measurements

Supernatants of differentiated CD8^+^ T cells were collected after culturing 5 × 10^6^ ml^−1^ cells in six-well plates for 24 h with IL-2 or IL-2+IL-4 in the absence or presence of 1,25D3 (100 nM or 1 μM). Pregnenolone levels were measured using the pregnenolone ELISA kit (ALPCO Diagnostics, Salem, NH) following the manufacturer’s protocol^4^.

### Immunoblot analyses

CD8^+^ T cells (5 × 10^6^) were lysed with Radioimmunoprecipitation assay buffer containing Halt protease and phosphatase inhibitor cocktail (Thermo Scientific, Rockford, IL) on ice for 30 min^4^,^27^. Samples were run by sodium dodecyl sulfate polyacrylamide gel electrophoresis (SDS–PAGE) and transferred to nitrocellulose membranes. The membranes were blocked using buffer containing 2% BSA and 0.5% sodium azide in Tris-Buffered Saline and Tween 20 for 1 h and incubated with rabbit polyclonal CYP11A1 antibody (Lifespan Biosciences, Seattle, WA) overnight at 4 °C. Horseradish peroxidase-conjugated anti-rabbit IgG (GE Healthcare, Hertfordshire, UK) was used to detect CYP11A1 protein. Mouse monoclonal anti-β-actin antibody (Sigma-Aldrich) was used as an internal control. Immunoreactive bands were quantified by densitometric quantification of autoradiographs using Image J (NIMH, Bethesda, MD), and expressed as relative CYP11A1 normalized to β-actin (Sigma-Aldrich).

### Chromatin immunoprecipitation

ChIP assays of cultured CD8^+^ T cells (5 × 10^6^) with IL-2 or IL-2+IL-4 in the presence or absence of 100 nM or 1 μM 1,25D3 were performed according to the manufacturer’s protocols using the ChIP IT express kit (Active Motif, Carlsbad, CA). Briefly, chromatin was crosslinked for 7 min at room temperature by adding 1% methanol-free formaldehyde (Thermo Fisher, Waltham, MA). Crosslinking was stopped by the addition of 1 × glycine-stop fix solution at room temperature. Following incubation of cells for 30 min at 4 °C, cell nuclei were pelleted and resuspended in 130 μl shearing buffer containing protease inhibitors. Shearing was conducted using an optimized 23-cycle treatment with a focused energy isothermal sonicator (Covaris S2 sonicator, Woburn, MA) leading to an average size of 300–500 bp, with highest density at 500 bp. Supernatant (10 μl) was saved for use as total input DNA.

Immunoprecipitations were performed according to the manufacturer’s protocols with protein G magnetic beads overnight at 4 °C with an estimated 2 μg sheared chromatin. Equal amounts of chromatin were used for the immunoprecipitation with the VDR-specific antibody (ab3508, Abcam, Cambridge, MA). After washing and eluting the beads with respective kit buffers, reverse crosslinking was performed for 15 min at 95 °C followed by a proteinase K treatment (10 μg ml^−1^) for 1 h at 37 °C. The immunoprecipitated genomic DNA was purified using the Qiagen PCR purification kit (Qiagen) and analysed via SYBR green (SYBR green Select Mastermix, Life Technologies) qPCR.

*In silico* transcription factor-binding analyses were performed using MatInspector (Genomatrix, Munich, Germany) and Alibaba2.2 (http://www.gene-regulation.com) to identify putative *VDR*-binding sites in the *Cyp11a1* promoter region. Respectively, *Cyp11a1* promoter-specific primers covering *VDR*-binding sites were designed using the VectorNTI 11 software (Supplementary Table 5). qPCR data were analysed via the percent input methodology: (2^CT of total input−CT of specific IP^) × 100 and relative fold change to percent input ratios using CD8^+^ T cells stimulated with IL-2 as baseline.

### Flow cytometry analyses

For intracellular staining, 1 × 10^6^ ml^−1^ cells were washed twice with PBS containing 1% BSA, stimulated with 1 μg ml^−1^ SIINFEKL in the presence of 2 μM monensin at 37 °C for 4 h. After fixation with 4% paraformaldehyde (Electron Microscopy Sciences, Hatfield, PA) and permeabilization with 0.1% saponin (Sigma-Aldrich), cells were washed twice with PBS containing 1% BSA, then incubated with anti-mouse CD16/CD32 (0.5 mg ml^−1^, 2 μl, 2.4G2, BD Bioscience, San Jose, CA) at 4 °C for 5 min, then stained with FITC labelled anti-mouse IFN-γ (0.5 mg ml^−1^, 5 μl, XMG 1.2, eBioscience, San Diego, CA) and PE-labelled anti-mouse IL-13 (0.5 mg ml^−1^, 5 μl, eBio13A, eBioscience). Cell staining was monitored on a FACSCalibur (BD Bioscience) and analysed using Flowjo software (Tree Star, Inc, Ashland, OR)^4^,^6^,^27^,^56^.

### Secondary allergen challenge model and adoptive transfer

The experimental protocol for sensitization and challenge to OVA was performed as described previously^4^,^6^,^27^,^56^,^57^ with some modifications. CD8-deficient mice were sensitized with 20 μg of OVA (Calbiochem) emulsified in 2.25 mg of alum (AlumImuject; Pierce, Rockford, IL) on days 0 and 14 by intraperitoneal injection. Sensitized mice were challenged with 0.2% OVA for 20 min on days 28, 29 and 30 using an ultrasonic nebulizer (model NE-U07; Omron Healthcare, Kyoto, Japan). To address the effect of 1,25D3 on CD8^+^ T-cell-mediated AHR, CD8^+^ T cells (5 × 10^6^) generated in medium containing IL-2 without or with 1,25D3 (100 nM or 1 μM) were injected into naive or OVA sensitized CD8-deficient mice intravenously on day 44. Two hours after cell transfer, naive and sensitized mice were re-challenged (secondary) with 1% OVA for 20 min by nebulization. Airway function was measured and samples were collected 48 h after the secondary challenge.

For some ChIP experiments, CD8^+^ T cells cultured in the presence of IL-2 without or with 1 μM 1,25D3 and adoptively transferred into sensitized and challenged recipients were isolated from the lungs. T cells were isolated from the lung after lung digestion^27^,^56^ with magnetic-activated cell sorting beads by positive selection using CD8^+^(Ly-2) microbeads (Miltenyi Biotec, San Diego, CA) providing 99% CD8^+^ T cells^27^,^56^.

### Assessment of airway function

Airway function was assessed as described previously^6^,^56^ by measuring changes in airway resistance (RL) in response to increasing doses of inhaled methacholine (MCh, Sigma-Aldrich). Data were presented as percentage change from the baseline RL values after saline inhalation. Baseline RL values were not significantly different among the various groups.

### BAL fluid analyses

After measurement of AHR, lungs were lavaged via the tracheal tube with 1 ml of Hanks' Balanced Salt Solution. The supernatants were collected and IL-4, IL-5 and IL-13 (eBiosicence, San Diego, CA) levels were measured by enzyme-linked immunosorbent assay (ELISA) following the manufacturer’s protocols^6^,^56^. The limits of detection for IL-4, IL-5 and IL-13 are 4 pg ml^−1^. Cytospin slides of leukocytes were stained with Leukostat (Fisher Diagnostics) and differentiated by standard haematological procedures in a blinded fashion^6^,^56^.

### Lung histology

Lungs were fixed in 10% formalin, and then embedded in paraffin. Paraffin sections (5 μm thick) were stained with periodic acid-Schiff (PAS). Mucus-containing goblet cells were quantified. Histology analysis was done in a blinded manner under light microscopy linked to an image system. The number of PAS-positive goblet cells was determined in cross-sectional areas of the airway wall. Six to ten different sections were evaluated per animal. The obtained measurements were averaged for each animal and the mean values and s.e.m. were determined for each group^4^,^27^.

### Statistical analyses for *in vitro*/*in vivo* mouse experiments

For outcome variables with multiple measures within mice (either within or between treatments), linear mixed models were employed, using the best available covariance structure for repeated measures; for outcome variables with one measure per mouse, general linear models were used, allowing separate variances by treatment (that is, weighted least squares). Pairwise comparisons were performed using *t*-tests derived from these models. Planned tests (five for each outcome) for AHR, differential cell count, BAL cytokine measurements, and lung histology (PAS) included comparisons using sensitized and challenged CD8-deficient mice either before or after adoptive transfer of untreated CD8^+^ T cells as baseline. For gene expression, CYP11A1 western blot, pregnenolone and ChIP experiments, CD8^+^ T cells differentiated either with IL-2 alone or in the presence of IL-2+IL-4 were used as baseline for all pairwise comparisons (seven tests for each outcome). The Benjamini–Hochberg^58^ procedure was used for the set of five or seven comparisons within each outcome variable to help control for false-positive test results, using a false discovery rate of 0.05.

Data for each outcome variable were based on at least 3 independent experiments, with multiple mice per treatment in each experiment. For the purposes of the analyses, data from each experiment were pooled together. Analyses for BAL and cytokine levels utilized a minimum of six mice per treatment, with unique mice across treatments; experiments for ChIP, gene expression, western blot and pregnenolone variables used a minimum of three independent experiments per treatment, with repeated measures across treatments; for lung histology (PAS), three to five mice per treatment were used, with independent mice across treatments, but where mice had multiple measurements within treatments (up to 9). *In vivo* experiments including AHR were performed with 11–13 mice per treatment group. For gene expression, western blot, pregnenolone and ChIP analyses, mice had repeated measures across different treatments. For the outcome variables in these analyses, a banded main diagonal structure was used that allowed for separate variances by treatment and no covariances between treatments; random intercepts for mice were also included in these models to account for within-mice correlation, but were excluded from final models since they did not improve model fits. For lung histology (PAS), mice had repeated measures within but not across treatments. For this outcome variable, a compound symmetric structure was used, allowing a separate variance parameter for the control treatment (sensitized and challenged CD8-deficient mice) relative to the other groups to account for the much lower variability for that condition. Results were expressed as the mean±s.e.m.

### Human study populations and *CYP11A1* and *VDR* genetic analyses

Based on the linkage disequilibrium (*r*^2^≥0.8, HapMap database, release #28, CEU population) within the *CYP11A1* locus (10 kb up- and 5 kb down-stream, Supplementary Fig. 4), we identified 25 *CYP11A1* SNPs with a minor allele frequency (MAF) ≥0.03 and two SNPs leading to an amino-acid change (rs6161 and rs1130841, MAF=0.005, Supplementary Fig. 6, black box). These *CYP11A1* polymorphisms clustered in five tagging bins and three single SNPs (Supplementary Fig. 5). Genotyping of rs9806234 failed but for the association analyses an imputation based data set originated from HapMap II was available^33^. None of the SNPs deviated from Hardy–Weinberg equilibrium (Table 1). No significant associations with asthma were observed for rs6161 (MAF=0.004, *n*=11 heterozygous subjects, five cases, six controls) and rs1130841 was monomorphic in the cohort under study. Thus, these mutations were not studied further.

Most of the asthmatics (*n*=655) for the association study included children of German or Austrian origin from the Multicenter Asthma Genetic In Childhood (MAGIC) study (mean age of 11±2.9 years s.d.)^33^,^59^,^60^ and 73 asthmatics and 767 healthy controls were derived from the International Study of Asthma and Allergies in Childhood (ISAAC) Phase II (*n*=5,629, mean age of 9.6±0.6 years s.d.)^61^. As previously described^60^, the subset of asthmatics was combined with asthmatics from the MAGIC study to test for associations in a case–control setting. No significant demographic differences between the populations under study were observed^60^. Genotyping of *CYP11A1* was performed by Illumina HumanHap300Chip (rs2279357, rs11632698, rs2073475, rs4432229, *N*=1,311)^33^,^59^ or by matrix-assisted laser desorption/ionization time-of-flight (MALDI-TOF) mass spectrometry (rs1484215, rs16968478, rs4432229, rs9806234, *n*=1,454, Table 1)^33^,^62^. Detailed information on the case–control data set and genotyping methodology including oligonucleotide sequences are available from the authors on request.

Genotyping data for each of the SNPs under study can be accessed using the following link http://www.share.asthmagene.org/permanentDownload/natComm15 (Phenotype: 1=asthma, 0=healthy control, Genotype: 0=homozygous wild-type allele, 1=heterozygous, 2=homozygous polymorphic allele). To comply with patient confidentiality, displayed ID numbers were randomly assigned for each SNP independently.

Deviation from Hardy–Weinberg equilibrium was analysed by *χ*^2^-test. Associations of binary traits were evaluated by logistic regression (Plink, version 1.07: http://pngu.mgh.harvard.edu/purcell/plink). Odds ratios (ORs), 95% confidence intervals (CIs) and *P* values are reported. Adjustment for multiple testing for the association analyses for *CYP11A1* SNPs was performed taking the LD into account. The effective number (*M*_eff_) of independent SNPs was calculated (SNPSpD (http://gump.qimr.edu.au/general/daleN/SNPSpD)^63^ to control for the experiment-wise significance level with an adjusted *P* value of *P*=0.010 (refs 63, 64). Selection for SNPs within *VDR* for gene-by-gene-interaction analyses with *CYP11A1* polymorphisms was based on previously studied *VDR* SNPs^33^.

All study subjects from both populations were of German descent. Written informed consent was obtained from all parents of children included in these studies. Study methods that were very similar in both populations were approved by the ethics committees of each of the study centres (ISAAC II: Dresden and Munich; MAGICS: Bochum, Cologne, Feldkirch, Freiburg, Munich/Rosenheim, Vienna and Wesel).

### *In silico* analyses of *CYP11A1* SNPs

Functional annotation was performed of the tagging and tagged *CYP11A1* SNPs associated with asthma. Transcription factor-binding analyses depending on the genotype status were conducted by Matinspector (www.genomatix.de) and Alibaba (www.gene-regulation.com). The putative regulatory role was further evaluated by the regulomeDB^65^. The regulomeDB score and potential transcription role of histone modifications in naive CD8^+^ T cells isolated from peripheral blood is presented. Allele-specific analyses on *CYP11A1* gene expression in EBV-transformed lymphoblastoid cell lines of unrelated samples of the HapMap population were performed (http://app3.titan.uio.no/biotools).

## Additional information

**How to cite this article:** Schedel, M. *et al*. 1,25D3 prevents CD8^+^Tc2 skewing and asthma development through VDR-binding changes to the *Cyp11a1* promoter. *Nat. Commun.* 7:10213 doi: 10.1038/ncomms10213 (2016).

## Supplementary Material

Supplementary Information Supplementary Figures 1-6 and Supplementary Tables 1-5

## Figures and Tables

**Table 1:** Asthma associations for *CYP11A1* SNPs in the case–control study population.

SNP	Allele*	MAF	Call rate (%)	LD bin	*P* value (HWE†)	Asthma (*n*=763)OR‡ (95% CI) *P* value
rs2279357**	G/A	0.28	99.7	5	0.64	0.85 (0.71–1.00) *P*=0.0554
rs6161	G/A	0.004	98.6		1.00	
rs11632698**	C/T	0.40	99.9	4	0.42	0.85 (0.73–1.00) *P*=0.0496
rs1484215	G/A	0.08	98.1	3	0.58	1.00 (0.76–1.32) *P*=0.9865
rs1130841	G/A	0	98.3			
rs2073475**	G/A	0.14	98.9	2	0.26	0.98 (0.78–1.23) *P*=0.8513
rs16968478	T/C	0.17	97.6		1.00	0.94 (0.77–1.14) *P*=0.5293
rs9806234§	T/C	0.26	100		0.76	0.99 (0.84–1.18) *P*=0.9478
rs4886595	T/G	0.19	97.3		0.91	0.77 (0.64–0.93) *P*=0.0079||
rs4432229**	T/C	0.16	99.9	1	0.49	0.80 (0.65–0.98) *P*=0.0351

CI, confidence interval; HWE, Hardy–Weinberg Equilibrium; LD, linkage disequilibrium; MAF, minor allele frequency; OR, odds ratio.

Significant associations in the case–control population (*N*=1,454 or *N*=1,311**) are marked in bold letters.

The statistical analyses were performed by logistic regression modelling additive effects. To control for the experiment-wise significance level, an adjusted *P* value of *P*=0.010 was used.

^*^Allele: wild-type/polymorphic allele.

^†^*P* value of *χ*^2^-test for deviation of HWE in controls.

^‡^OR and 95% CI: polymorphic (minor) allele used as basis for the calculation of the effect size.

^§^Data obtained from imputed data set.

^||^Association between rs4886595 and asthma remains significant after correction for multiple testing with an adjusted *P* value of 0.010.

**Table 2:** Epistatic effects on asthma susceptibility of polymorphisms in *CYP11A1* and *VDR*.

Effect of	In individuals carrying	Reference allele*	Odds ratio (95% CI)	*P* value
*CYP11A1* rs4886595		G	0.77 (0.64–0.93)	**0.0079**
*CYP11A1* rs4432229		C	0.80 (0.65–0.98)	**0.0351**
*VDR* rs2107301		A	0.76 (0.64–0.89)	**0.0010**
*CYP11A1* rs4886595	*VDR* rs2107301 GG (*n*=660)	*G*	0.71 (0.54–0.94)	**0.0176**
*CYP11A1* rs4886595	*VDR* rs2107301 GA+AA (*n*=608)	*G*	0.82 (0.62–1.10)	0.1894
*VDR* rs2107301	*CYP11A1* rs4886595 TT (*n*=835)	*A*	0.75 (0.60–0.93)	**0.0082**
*VDR* rs2107301	*CYP11A1* rs4886595 TG+GG (*n*=433)	*A*	0.73 (0.54–0.98)	**0.0372**
*CYP11A1* rs4432229	*VDR* rs2107301 GG (*n*=678)	*C*	0.73 (0.54–0.98)	**0.0354**
*CYP11A1* rs4432229	*VDR* rs2107301 GA+AA (*n*=624)	*C*	0.90 (0.67–1.21)	0.4785
*VDR* rs2107301	*CYP11A1* rs4432229 TT (*n*=928)	*A*	0.75 (0.61–0.91)	**0.0051**
*VDR* rs2107301	*CYP11A1* rs4432229 TC+CC (*n*=374)	*A*	0.76 (0.56–1.05)	0.0970

The entire case–control study population was stratified for the *CYP11A1* polymorphisms rs4886595, and rs4432229 and rs2107301 (*VDR*) to investigate the SNP effect on asthma susceptibility of one SNP in relation to the other polymorphism. Significant associations are marked in bold letters. The statistical analyses were performed by logistic regression modelling additive effects.

^*^Allele: polymorphic (minor) allele was used as basis for the calculation of the effect size.
