# Supplementary material for: 1,25D3 prevents CD8+Tc2 skewing and asthma development through VDR binding changes to the Cyp11a1 promoter
Source: Nat Commun. 2016 Jan 11;7:10213. doi: 10.1038/ncomms10213 (PMC4712703; doi:10.1038/ncomms10213)
Supplement: Supplementary Information — Supplementary Figures 1-6 and Supplementary Tables 1-5 [file ncomms10213-s1.pdf]

Supplementary Figure 1

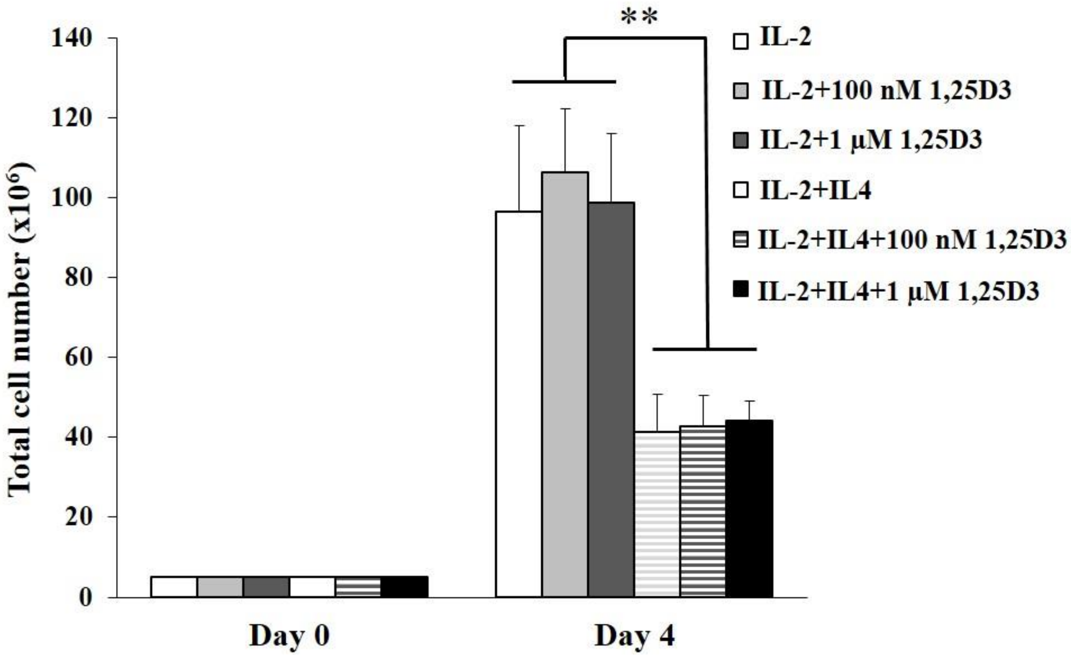

Cell viability of CD8+ T cells differentiated in IL-2 or IL-2+IL-4 is not altered by 1,25D3 (100 nM or 1 μM). Total cell numbers were counted on days 0 and 4 during CD8+ T cells differentiation in IL-2 or IL-2+IL-4 in the presence or absence of 100 nM or 1 μM 1,25D3. Data (mean±SEM) are from five experiments. **p<0.01 compared to the IL-2 group on day 4.

Supplementary Figure 2

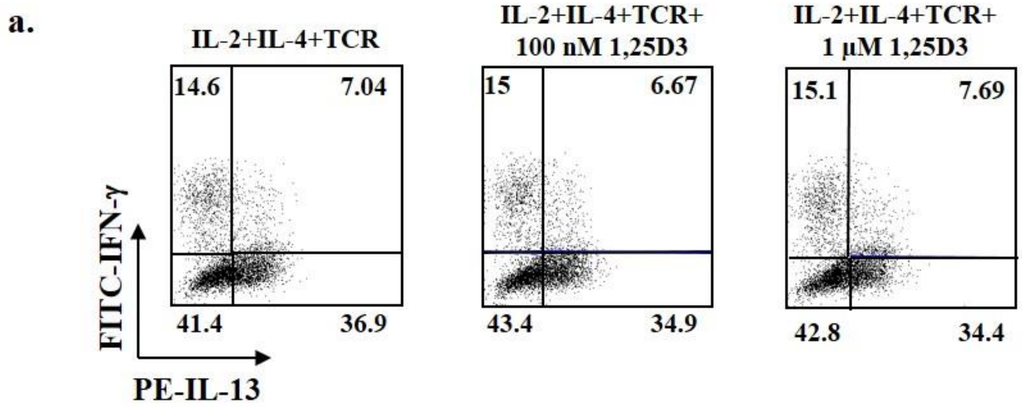

b.

**Groups**	**IL-2+IL-4+ TCR**	**IL-2+IL-4+ TCR+ 100 nM 1,25D3**	**IL-2+IL-4+ TCR+ 1 µM 1,25D3**
**IFN-γ$^+$**	14.5±0.3	14.7±0.3	15.1±0.4
**IL-13$^+$**	31.1±3.6	30.2±2.6	28.7±3.2
**IFN-γ$^+$/IL-13$^+$**	4.6±1.4	4.6±1.2	4.6±1.7
**IFN-γ$^-$/IL-13$^-$**	49.8±5	52.5±6.8	51.6±4.9

Presence of 1,25D3 only during the antigen re-stimulation phase does not alter IFN-γ and IL-13 production in CD8$^+$ T cells differentiated in IL-2 or IL-2+IL-4. (a) Representative results of intracellular staining and **(b)** data (mean±SEM) showing percent positive cells (four independent experiments) for IFN-γ and IL-13 expression in CD8$^+$ T cells differentiated in IL-2 or IL-2+IL-4 with SIINFEKL (T cell receptor, TCR) restimulation. 1,25D3 (100 nM or 1 µM) was added for 4 hours only during the antigen restimulation phase.

Supplementary Figure 3

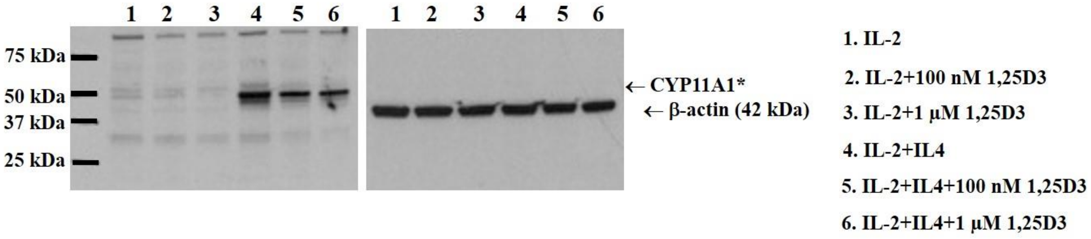

1,25D3 treatment of CD8$^+$ T cells alters CYP11A1 protein levels. Representative results of a CYP11A1 western blot of CD8$^+$ T cells differentiated in IL-2 or IL-2+IL-4 in the presence of absence of 100 nM or 1 µM 1,25D3. * calculated molecular weight (MW) for CYP11A1 (13363-1-AP, Proteintech, Chicago, IL): 60 kDa, observed MW: 49 kDa

Supplementary Figure 4

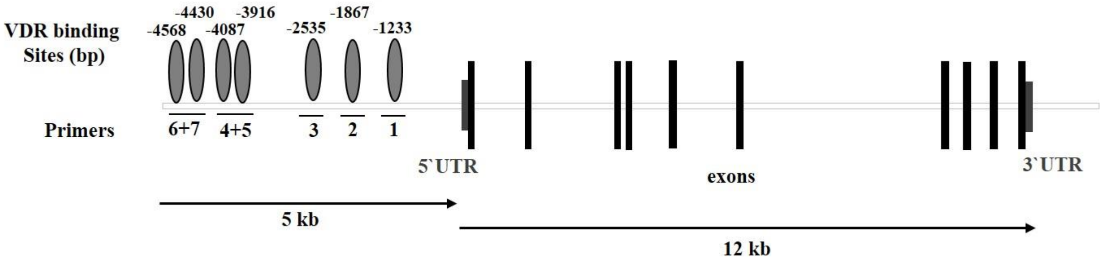

Genomic structure of *Cyp11a1* and localization of VDR binding sites in the *Cyp11a1* promoter region. Exons are displayed in black and smaller grey boxes represent 5` and 3` untranslated regions (UTR). VDR binding sites in the *Cyp11a1* promoter and respective *Cyp11a1*-specific primers are depicted relative to the location of the CYP11A1 coding region.

Supplementary Figure 5

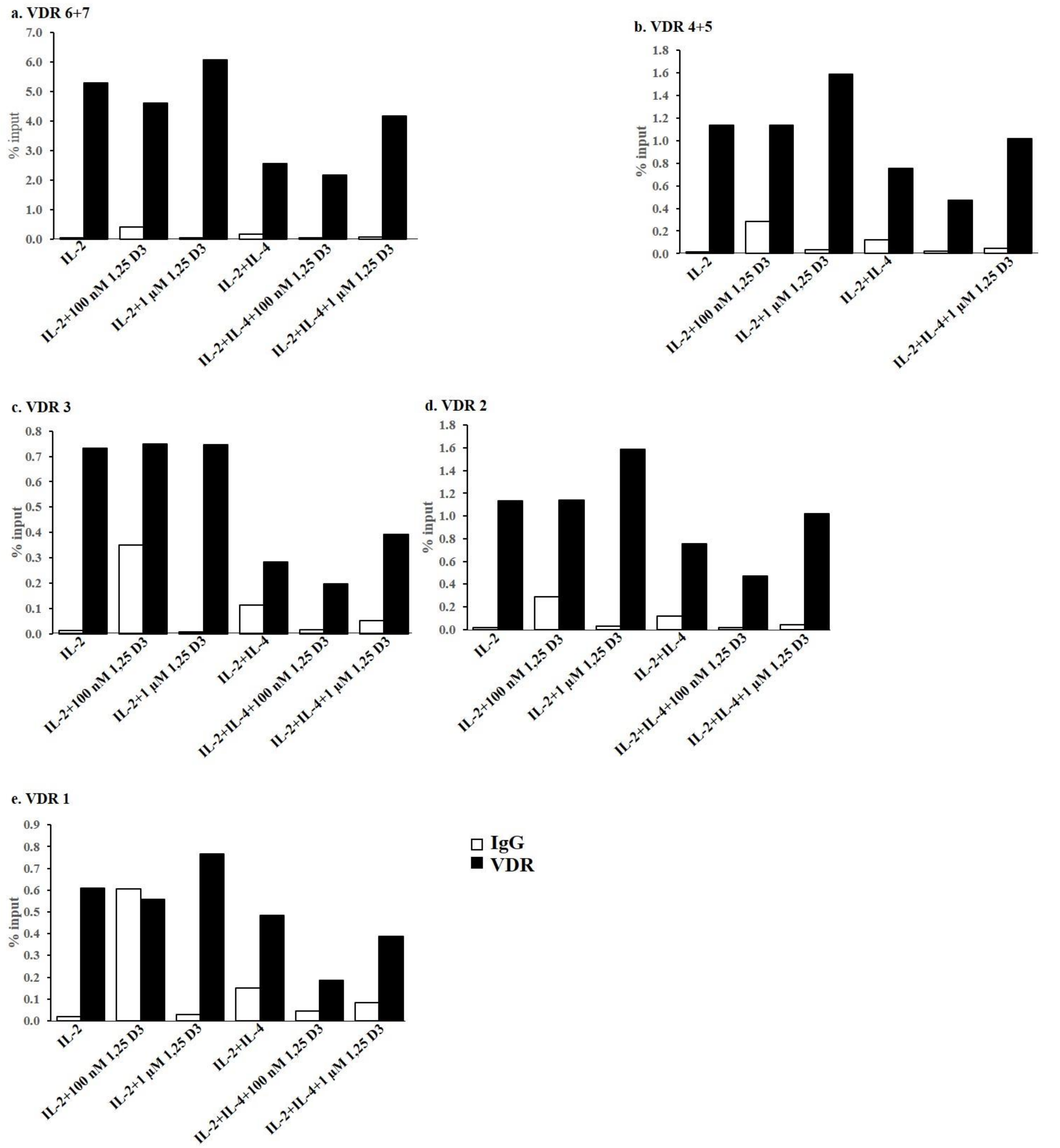

1,25D3-mediated VDR recruitment to the *Cyp11a1* promoter region is altered in CD8$^+$ T cells differentiated in IL-2 or IL-2+IL-4 in the presence or absence of 100 nM or 1 µM 1,25D3. (a-e) A representative experiment of the percent input immunoprecipitated by the VDR and the negative control IgG antibody. qPCR was performed using 5 *Cyp11a1* promoter-specific primers [**(a)** VDR 6+7, **(b)** VDR 4+5, **(c)** VDR 3, **(d)** VDR 2, **(e)** VDR1] covering seven VDR binding sites. Data were analyzed via the percent input methodology: (2^ (CT of total input – CT of specific IP)) x100.

Supplementary Figure 6

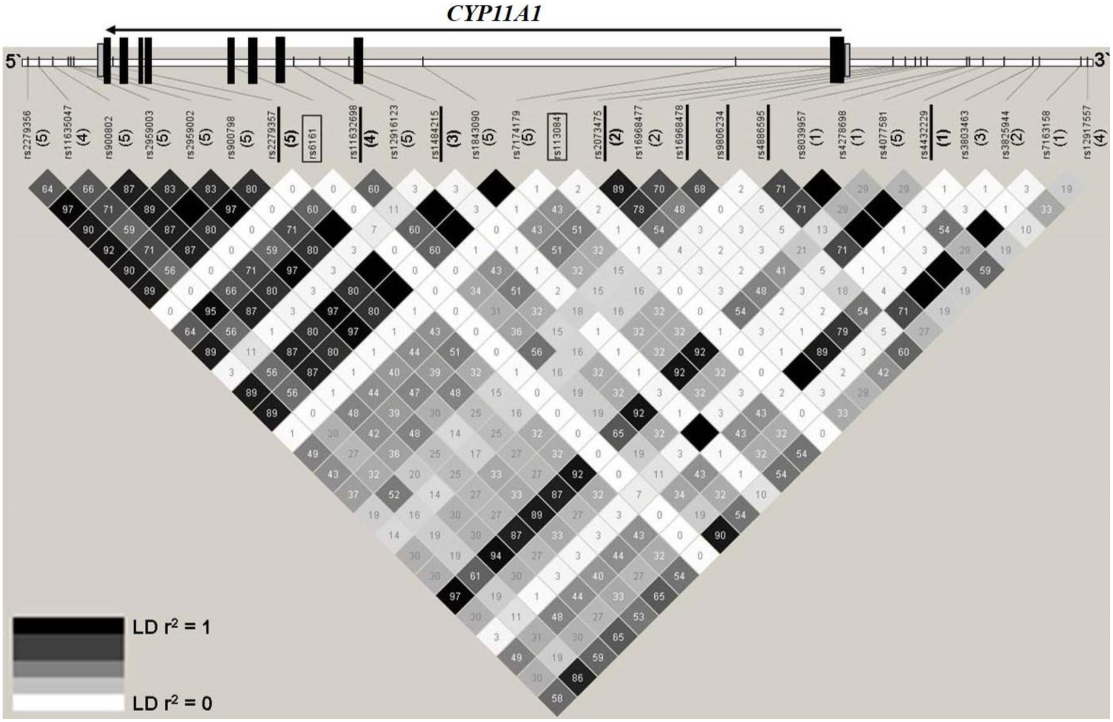

Genomic structure, localization, and linkage disequilibrium (r^2 plot) of the genotyped *CYP11A1* polymorphisms. In the upper panel, the genomic structure and localization of the genotyped tagging polymorphisms within the *CYP11A1* locus are depicted. Arrows indicate transcriptional direction. Exons are displayed in black and smaller grey boxes represent 5` and 3` untranslated regions (UTR). In the lower panel, linkage disequilibrium (r^2 plot) corresponding to the respective tagging bins (bins 1-5) of *CYP11A1* is shown with $r^2>0.80$. Tagging SNPs (MAF≥0.03) are underlined. In addition, rs6161 and rs113084 (MAF≤0.005) were genotyped as they lead to an amino acid change (marked in black box).

Supplementary Table 1: IFN-γ and IL-13 expression in $CD8^+$ T cells differentiated in IL-2 or IL-2+IL-4 in the presence of 1,25D3.

	% of positive cells											
	**IL-2**	**IL-2+ VitD (100nM)**	**IL-2+ VitD (1µM)**	**IL-2+ IL4**	**IL-2+ IL-4+ VitD (100nM)**	**IL-2+ IL-4+ VitD (1µM)**	**IL-2+ TCR**	**IL-2+ TCR+ VitD (100nM)**	**IL-2+ TCR+ VitD (1µM)**	**IL-2+ IL-4+ TCR**	**IL-2+ IL-4+ TCR+ VitD (100nM)**	**IL-2+ IL-4+ TCR+ VitD (1µM)**
**IFN-γ^+**	3.3 ±2	3.1 ±1.3	4.3 ±2.1	1.6 ±0.8	2.2 ±1	2.7 ±1.2	79.8 ±6.6	76.8 ±5.3	76.2 ±5.1	16.8 ±5.6	24.5 ±4.8	30.6 ±5
**IL-13^+**	0.4 ±0.2	0.2 ±0.1	0.2 ±0.1	9.7 ±2.9	4.6 ±1.1	3.8 ±0.9	0.2 ±0.3	0.1 ±0.2	0.1 ±0.1	23.8 ±9.3	11.3 ±4.8	8.3 ±3.7
**IFN-γ^+ IL-13^+**	0.1 ±0.1	0	0	0.2 ±0.2	0.2 ±0.1	0.2 ±0.2	0.7 ±0.7	0.4 ±0.5	0.4 ±0.3	5.8 ±1.9	4 ±1.3	3.5 ±1.2
**IFN-γ^- IL-13^-**	96.2 ±2	96.8 ±1.3	95.8 ±2.1	88.4 ±3.2	92.7 ±1.3	92.9 ±1.3	20.1± 5.8	22.6 ±4.3	23.7 ±4.5	48.9 ±6.9	55.1 ±8.9	53.9 ±6.5

Supplementary Table 2: Description of *CYP11A1* polymorphisms and their respective rs numbers, position within the gene, allele frequencies, linkage disequilibrium and genotyped tagging SNPs.

**rs number**	**Position in the gene structure**	**Minor allele frequency (MAF)[1]**	**LD[2] (r^2) with tagging SNP**	**Tagging SNP**	**Tagging block**
rs12917557	promoter	0.45	0.90	rs11632698	4
rs7163158	promoter	0.11	1.0	rs4432229	1
rs3825944	promoter	0.17	1.0	rs2073475	2
rs3803463	promoter	0.07	1.0	rs1484215	3
**rs4432229**	promoter	**0.11**			**1**
rs4077581	promoter	0.28	0.92	rs2279357	5
rs4278698	promoter	0.11	1.0	rs4432229	1
rs8039957	promoter	0.11	1.0	rs4432229	1
rs4886595	promoter	0.14			
rs9806234	promoter	0.27			
rs16968478	promoter	0.21			
rs16968477	promoter	0.16	0.89	rs2073475	2
**rs2073475**	promoter	**0.18**			**2**
rs1130841	exon 1	0.005			
rs7174179	intron 1	0.27	1.0	rs2279357	5
rs1843090	intron 1	0.27	1.0	rs2279357	5
**rs1484215**	intron 2	**0.08**			**3**
rs12916123	intron 2	0.27	1.0	rs2279357	5
**rs11632698**	**intron 2**	**0.37**			**4**
rs6161	exon 5	0.005			
**rs2279357**	**intron 8**	**0.27**			**5**
rs900798	downstream	0.30	0.80	rs2279357	5
rs2959002	downstream	0.27	0.97	rs2279357	5
rs2959003	downstream	0.30	0.80	rs2279357	5
rs900802	downstream	0.29	0.87	rs2279357	5
rs11635047	downstream	0.37	0.95	rs11632698	4
rs2279356	downstream	0.27	0.89	rs2279357	5

[1] Minor allele frequency (MAF) = based on the HapMap CEU population

[2] LD = linkage disequilibrium

Supplementary Table 3: *In silico* analyses for putative regulatory functions for asthma-associated C*YP11A1* SNPs.

Tagging bin	SNP	Location	Major allele[1]	Minor allele[1]	Major allele[2]	Minor allele[2]	RegulomeDM Score[3]	Histone modifications[3]	beta-score (p-value)[4]
1	rs8039957	promoter	SP1	SP1	3	8	6	weak Repressed PolyComb	-0.04 (0.0780)
	rs4278698	promoter	SP1, Tra-1	REV-Erb A	5	4	no data	no data	-0.04 (0.1380)
	**rs4432229**	promoter	CEBPα	HNF1C	4	6	4	active TSS	-0.04 (0.0780)
	rs7163158	promoter	REV-Erb A, COUP	REV-Erb A, COUP, ARP1, SP1	16	18	6	weak transcription	-0.06 (0.0223)
	**rs4886595**	promoter	CEBPα, -β, Oct-1, NF-EM5, MyoD	CEBPα, -β, Oct-1	10	13	3a	quiescent/low	-0.06 (0.0028)
2	rs12917557	promoter	NF-κB, PTF1β, SP1	NF-κB, PTF1β, GCN4, SP1	6	7	5	quiescent/low	-0.01 (0.6262)
	**rs11632698**	intron 2	GR, CEBPα	CEBPα	13	17	2a	quiescent/low	-0.04 (0.1380)
	rs11635047	downstream					5	quiescent/low	0.01 (0.7371)

SNPs in bold letters indicate tagging SNPs

[1]Different predicted transcription factor (TF) binding sites depending on the genotype using Matinspector (www.genomatix.de).

[2]Different predicted TF binding sites depending on the genotype using Alibaba (www.gene-regulation.com).

[3]Putative regulatory function based on regulomeDB, predicted histone modifications are based on data available from naive primary $CD8^+$ cells from peripheral blood.

2a: TF binding + matched TF motif + matched DNase footprint + DNase peak

3a: TF binding + any motif + DNase peak

4: TF binding + DNase peak

5: TF binding + DNase peak

6: Other

[4]Allele-specific gene expression analyses in EBV-transformed lymphoblastoid cell lines of unrelated samples of the HapMap population (http://app3.titan.uio.no/biotools).

Supplementary Table 4: qPCR primers used for gene expression experiments.

Primer name	Sequence (5’3’)
Gata3 forward	GACTCTTCCCACCCAGCAGC
Gata3 reverse	CCATCTCGCCGCCACAG
Gata3 probe	CAAGGCACGATCCAGCACAG
Tbx21 (LifeTechnologies pre-designed assay)	Mm00450960_m1
Cyp11a1 forward	TGATGACCTATTCCGCTTTTCC
Cyp11a1 reverse	GGTTGAGCATGGGGACACTG
Cyp11a1 probe	ATGCTGGAGGAGATCGTGGA
Vdr forward	AGAAGGCTCCGATGACCCC
Vdr reverse	AAGGTAAAAGACTGGTTGGAGCG
Vdr probe	CCGCTCTCCATGCTGCCCCACC
18SrRNA forward	AGTCCCTGCCCTTTGTACACA
18SrRNA reverse	GATCCGAGGGCCTCACTAAAC
18SrRNA probe	CGCCCGTCGCTACTACCGATTGG

Supplementary Table 5: *Cyp11a1* promoter-specific primers used for VDR ChIP experiments.

Primer name	Primer sequence (5'3)
VDR_1fwd	CTGGGGCTACACCGTGATTC
VDR_1rev	CTTTTAAGATCCCCCTGCCTC
VDR_2fwd	CTTGCTAGAACCCAGTGTAATGAAC
VDR_2rev	AAGTTCACAGCGGTCCTCG
VDR_3fwd	GCATTGATCCCAGAGAGGTTAAG
VDR_3rev	CTGGTCCCATTGGCTTCTG
VDR_4+5fwd	GCTTCCTGAGTTGAGTTTTGTTATG
VDR_4+5rev	CCATCCCTCCCTCCAAGC
VDR_6+7fwd	TCGCCTGTCTCTGCCTCC
VDR_6+7rev	TGAGTTCTAGTCCCCAGCAGC
